# Simultaneous utilization of glucose, xylose and arabinose in the presence of acetate by a consortium of *Escherichia coli* strains

**DOI:** 10.1186/1475-2859-11-77

**Published:** 2012-06-12

**Authors:** Tian Xia, Mark A Eiteman, Elliot Altman

**Affiliations:** 1 Center for Molecular BioEngineering, Department of Biological and Agricultural Engineering, University of Georgia, Athens, GA 30602, USA; 2 Department of Biology, Middle Tennessee State University, Murfreesboro, TN, 37132, USA

**Keywords:** Lignocellulosic hydrolysate, Sugar mixtures, Growth inhibitors, Phosphotransferase system

## Abstract

**Background:**

The efficient microbial utilization of lignocellulosic hydrolysates has remained challenging because this material is composed of multiple sugars and also contains growth inhibitors such as acetic acid (acetate). Using an engineered consortium of strains derived from *Escherichia coli* C and a synthetic medium containing acetate, glucose, xylose and arabinose, we report on both the microbial removal of acetate and the subsequent *simultaneous* utilization of the sugars.

**Results:**

In a first stage, a strain unable to utilize glucose, xylose and arabinose (ALS1392, strain *E. coli* C *ptsG manZ glk crr xylA araA*) removed 3 g/L acetate within 30 hours. In a subsequent second stage, three *E. coli* strains (ALS1370, ALS1371, ALS1391), which are each engineered to utilize only one sugar, together simultaneously utilized glucose, xylose and arabinose. The effect of non-metabolizable sugars on the metabolism of the target sugar was minimal. Additionally the deletions necessary to prevent the consumption of one sugar only minimally affected the consumption of a desired sugar. For example, the *crr* deletion necessary to prevent glucose consumption reduced xylose and arabinose utilization by less than 15% compared to the wild-type. Similarly, the *araA* deletion used to exclude arabinose consumption did not affect xylose- and glucose-consumption.

**Conclusions:**

Despite the modest reduction in the overall rate of sugar consumption due to the various deletions that were required to generate the consortium of strains, the approach constitutes a significant improvement in any single-organism approach to utilize sugars found in lignocellulosic hydrolysate in the presence of acetate.

## Background

Lignocellulose is the most abundant source of biomass for the renewable production of fuels and chemicals, readily available from dedicated crops and agricultural, industrial, forestry and municipal residues [1]. Hydrolysis of lignocellulose results in a mixture of sugars including the hexoses D-glucose, D-galactose, and D-mannose, and the pentoses D-xylose and L-arabinose, and uronic acids [2]. The relative proportion of cellulose, hemicellulose and lignin varies widely among different biomass sources, as does the composition of the hemicellulose fraction itself. Although glucose is the most abundant hexose, and xylose is typically the principal pentose, the arabinose fraction found in hydrolysates can be significant depending on the materials and the process. For example, the liquor from the sulfite cooking of spruce was found to contain 34.3% arabinose, 25.5% xylose and 4.4% glucose [3]. Also, a dilute acid hydrolysate of sugar cane bagasse used for fermentation contained 75.7 g/L xylose, 13.5 g/L arabinose (with mannose) and 13.2 g/L glucose [4]. The microorganisms which are desirable for forming a product of interest such as ethanol generally do not utilize all of these sugars efficiently. For example, the widely used platform organism yeast *Saccharomyces cerevisiae* does not naturally consume pentoses as a carbon source. Even species such as *Escherichia coli* which metabolize all of these sugars suffer from glucose repression which often prevents pentose consumption in the presence of glucose.

Significant effort has focused on developing a single organism which can simultaneously consume the multiple sugars found in lignocellulosic hydrolysates. For example, three genes associated with xylose utilization when expressed in *S. cerevisiae* improve xylose utilization in that strain, although 75% of the xylose remains at the time glucose becomes depleted [5]. Additionally, expressing heterologous arabinose-metabolising enzymes in this yeast permits simultaneous glucose, xylose and arabinose utilization [6,7], although compared to glucose, the xylose consumption rate was more than ten times slower and arabinose consumption over twenty times slower. Moreover, sugar alcohols xylitol and arabitol often accumulate from pentose utilization [6]. Improvements to *E. coli*, which first metabolizes glucose in a mixture of glucose, xylose, and arabinose, initially focused on disrupting the normal phosphotransferase system (PTS) of glucose uptake. For example, pentose consumption is improved in the presence of glucose by a knockout of the *ptsG* gene encoding enzyme IICB^Glc^[8]. Similarly, a *ptsG* mutant strain IT1168 metabolized xylose and arabinose simultaneously with glucose, rather than using glucose preferentially [9]. Although the *ptsG* mutation improves xylose consumption, 40% of the xylose remained when the glucose was depleted [10].

An additional complication in the utilization of lignocellulosic hydrolysates is the presence of microbial inhibitors which reduce the cells' rate of sugar consumption. The most studied inhibitory compound is acetic acid, which is formed from biomass by the hydrolysis of acetylxylan, and which is present in hydrolysates at concentrations of generally 2–5 g/L [11,12]. A concentration of 8 mM (0.48 g/L) acetate reduced the growth rate of *E. coli* on sugars by 50% [13]. Interestingly, acetate itself is metabolizable, and substantial research has been conducted to understand the mechanism of acetate tolerance in *E. coli* and other organisms [14].

In addition to the specific (i.e., per cell) rate of pentose consumption being generally slower than the rate of glucose consumption, a fundamental disadvantage of any single-organism approach toward the utilization of multiple substrates is that a single organism has not yet been designed which adjusts its consumption rate to multiple carbon sources in proportion to their availability. For example, when a feed medium is step-changed to a xylose-glucose mixture having twice as much xylose as previously, a single microorganism is unable to consume xylose now twice as quickly.

A different approach for the conversion of multiple sugars into any product is to use a consortium of the *same* species of microorganism, with each member of that consortium designed to consume *only* one substrate [15]. When members of the consortium are the same species, growth incompatibilities (pH, temperature, nutritional requirements, negative cell-to-cell interactions) are avoided [16]. Each member of the consortium therefore effectively ignores other substrates while carrying out the one target conversion. One advantage demonstrated for this substrate-selective uptake is that the system can adapt to fluctuations in the feed stream; that is, culture populations change in concert with a variable feed composition, which was demonstrated for the conversion of a xylose and glucose mixture into lactic acid [17]. The same approach has been used to remove acetate selectively from sugars [18]. Presumably additional, targeted metabolic engineering strategies could be used to form other desired products independently from each of the strains making up the consortium.

The goal of this current study was to extend the consortium approach for mixed sugar utilization to a synthetic mixture composed of the three sugars glucose, xylose and arabinose as well as the inhibitor acetic acid. Two approaches for the consumption of acetate and three sugars were compared. In one case, an acetate-selective strain was first used to remove this compound from the mixture, and then with acetate exhausted, the three remaining sugars were simultaneously consumed by introducing a consortium of three substrate-selective strains into the culture. As a second approach, acetate removal was accomplished in conjunction with sugar consumption by using all four strains together. Finally, we examined the impact of the presence of multiple sugars on the consumption of each metabolizable sugar.

## Results

The concept proposed for the simultaneous utilization of the three principal sugars—glucose, xylose and arabinose—found in lignocellulosic hydrolysates relies on three strains which each can *only* metabolize one of these sugars. Because wild-type *E. coli* can utilize all three sugars, each strain to be constructed had to be *unable* to consume two of the three sugars. For the elimination of glucose consumption, the four genes *ptsG*, *manZ*, *glk* and *crr* play the most important roles in glucose uptake. The *ptsG* gene encodes the Enzyme IICB^Glc^ of the glucose PTS [19], and is quite specific for D-glucose. The *manZ* gene encodes the IID^Man^ domain of the mannose PTS [20], and the protein product is able to phosphorylate glucose and mannose, as well as their derivatives altered at the C2 position [21]. The *glk* gene encodes glucokinase [21], which phosphorylates glucose in the cytoplasm using ATP in strains lacking the PTS [22]. The *crr* gene encodes the IIA^Glc^ protein of the glucose PTS*,* and it serves a central role in carbon catabolite repression [23]. Mutants lacking both *ptsG* and *manZ* grow very slowly on glucose, while an *E. coli* K-12 strain additionally lacking glucokinase was previously reported to be unable to grow on glucose [21]. Deletions of genes *xylA* and *araA* encoding D-xylose isomerase and L-arabinose isomerase, the first metabolic steps in the metabolism of these pentoses, respectively, are known to eliminate xylose [24] and arabinose consumption [25]. We thus constructed strains ALS1370 (C *ptsG glk manZ crr xylA*), ALS1371 (C *ptsG glk manZ crr araA*) and ALS1391 (C *xylA araA*) which respectively are unable to metabolize xylose/glucose, arabinose/glucose and xylose/arabinose. Therefore, these strains selectively consume only arabinose (ALS1370), xylose (ALS1371) or glucose (ALS1391).

### Single sugar growth

When each of the three strains alone was grown in a medium containing the single metabolizable sugar, that sugar was depleted within 4 h, and the growth rates were all greater than 0.7 h^-1^ (Table 1). The growth rates of the substrate-selective strains ALS1370 and ALS1371 were 12–15% less than the growth rate of the wild type C strain on their respective sugar, whereas no difference was observed between the growth rates of the glucose-selective strain ALS1391 and the wild-type C (on glucose).

**Table 1:** **Maximum specific growth rates (h**^**-1**^**) of *E. coli *strains on different carbon sources**

		**Maximum Specific Growth Rate (h**^**-1**^**)**			
**Strain**	**Genotype**	**Arabinose**	**Xylose**	**Glucose**	**Three-Sugar**
ATCC8739	C	0.88	0.81	0.87	–
ALS1370	C *ptsG glk manZ crr xylA*	0.76	–	–	0.74
ALS1371	C *ptsG glk manZ crr araA*	–	0.72	–	0.55
ALS1391	C *xylA araA*	–	–	0.86	0.59

Each single-sugar medium contained the specified sugar (D-(+)-glucose, D-(+)-xylose, or L-(+)-arabinose) at a concentration of 2.25 g/L. The three-sugar mixture contained all three sugars at the same concentration. The standard deviation of the each measurement was about 0.01 h^-1^.

### Growth of each strain on a sugar mixture

**Figure 1:**
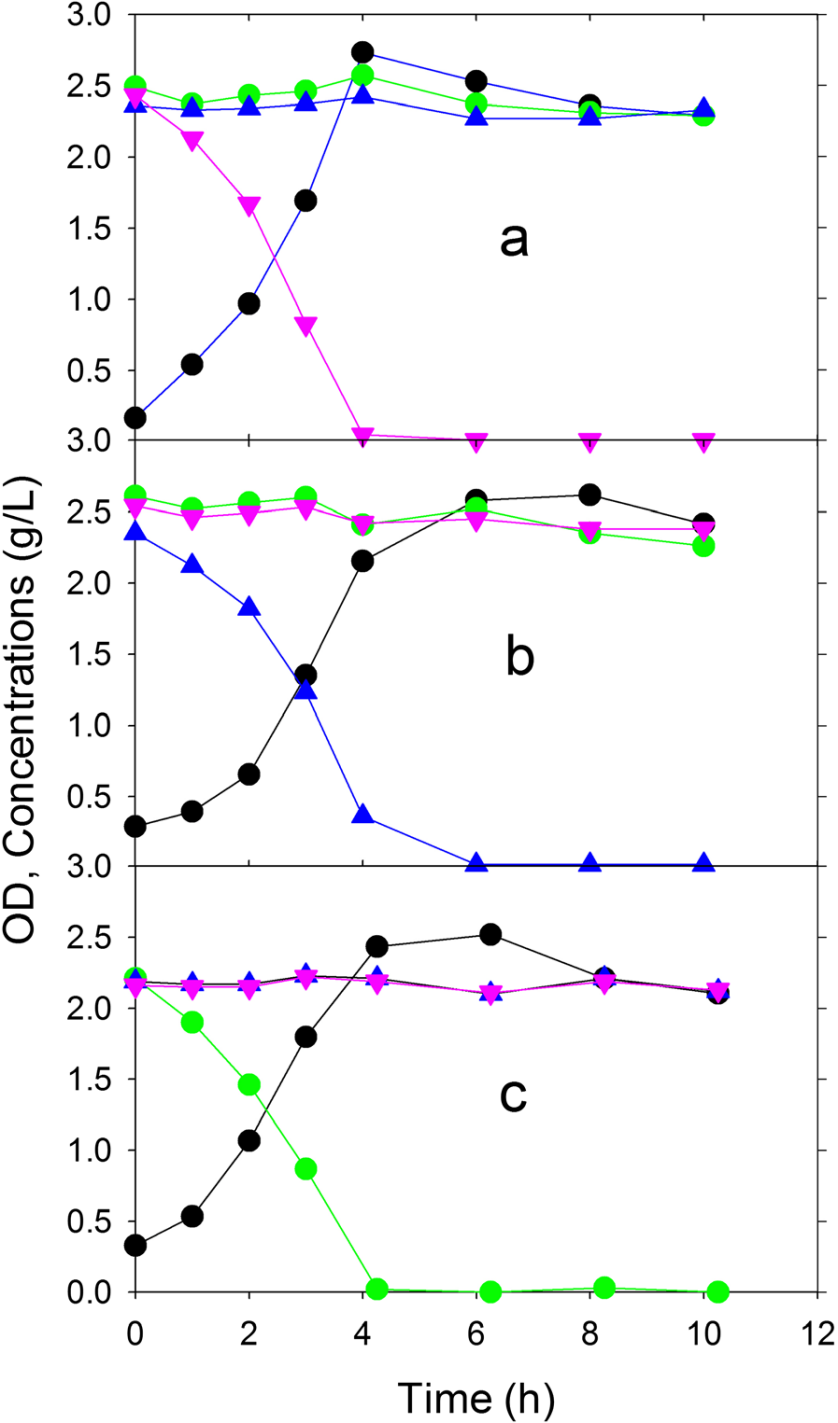
**Single strain growth in shake flasks containing a three sugar mixture (glucose, xylose and arabinose).** Growth (OD, *black circle*) of *E. coli* arabinose-selective strain ALS1370 (**a**), xylose-selective strain ALS1371 (**b**) or glucose-selective strain ALS1391 (**c**) separately in a medium containing three sugars: xylose (*blue triangle*), arabinose (*pink inverted triangle*) and glucose (*green circle*).

In order to determine whether the constructed knockouts were able to metabolize one sugar selectively in the presence of the other two sugars, each strain was cultivated in a simulated mixture of glucose, xylose and arabinose. Each strain successfully exhausted its target monosaccharide within 4–6 h without degradation of the other two sugars. The growth rates of ALS1371 (xylose) and ALS1391 (glucose) on the three-sugar mixtures were 20–30% lower than the growth rates on their corresponding single sugars, while the growth rate of ALS1370 was not significantly different on arabinose alone or arabinose in the three-sugar mixture (Table 1). Figure 1 shows the growth and substrate utilization for each strain alone in a mixture of 2.25 g/L sugars. For example, ALS1370 consumes arabinose completely after 4 h, but leaves xylose and glucose unutilized (Figure 1a). These experiments demonstrate that each strain grows on a single sugar without consuming the two other sugars.

The previous experiments were conducted by first growing the strains individually in a medium containing only the one metabolizable sugar, and using this culture to inoculate the three-sugar mixture. In order to examine whether each strain would adapt to the presence of the other two sugars, we also conducted the experiment using the three-sugar mixture in the inoculum. In these cases, the growth rates observed in the second experimental shake flask did not differ from the single-sugar inocula, and again in each case only the one intended sugar was consumed (data not shown).

Because the growth rates of ALS1371 and ALS1391 were significantly lower on the mixture of sugars than on either xylose or glucose alone, respectively, several additional experiments were completed. ALS1371, which consumes only xylose, was grown on the two-sugar mixtures of glucose-xylose and arabinose-xylose. On the mixture of glucose-xylose, ALS1371 had a maximum specific growth rate of 0.63 h^-1^, whereas on the mixture of arabinose-xylose ALS1371 had a maximum specific growth rate of 0.61 h^-1^. Similarly, the glucose-selective strain ALS1391 was grown on the two-sugar mixtures of glucose-xylose and glucose-arabinose. On the mixture of glucose-xylose, ALS1391 had a maximum specific growth rate of 0.70 h^-1^, whereas on the mixture of glucose-arabinose, ALS1391 had a maximum specific growth rate of 0.48 h^-1^.

### Consortium utilization of sugars

**Figure 2:**
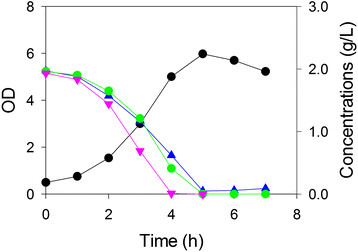
**Three strain growth in a shake flask containing a three sugar mixture (glucose, xylose and arabinose).** Growth (OD, *black circle*) of *E. coli* arabinose-selective strain ALS1370, xylose-selective strain ALS1371 and glucose-selective strain ALS1391 together in a medium containing three sugars: xylose (*blue triangle*), arabinose (*pink inverted triangle*) and glucose (*green circle*).

Since each of the three strains selectively utilized a single sugar from a three-sugar mixture, we were next interested in combining the three strains in the same mixture. Figure 2 shows the growth of the three strains *together* on the mixture of glucose, xylose and arabinose. The figure clearly shows that the three sugars are metabolized simultaneously without significant lag or inhibition. Each of the three sugars was exhausted in about 5 h, fairly close to the time observed for each strain growing alone on one sugar or on the sugar mixture (Figure 1). The increase in cell density for the consortium of microorganisms in this case represents the total population of all three strains. This “apparent growth rate” was about 0.65 h^-1^, roughly equal to the mean of the three growth rates found using single strains on sugar mixtures (0.63 h^-1^). Furthermore, the observed OD for the consortium growing on the sugar mixture was approximately the sum of the ODs of each strain growing on their one metabolizable sugar.

**Figure 3:**
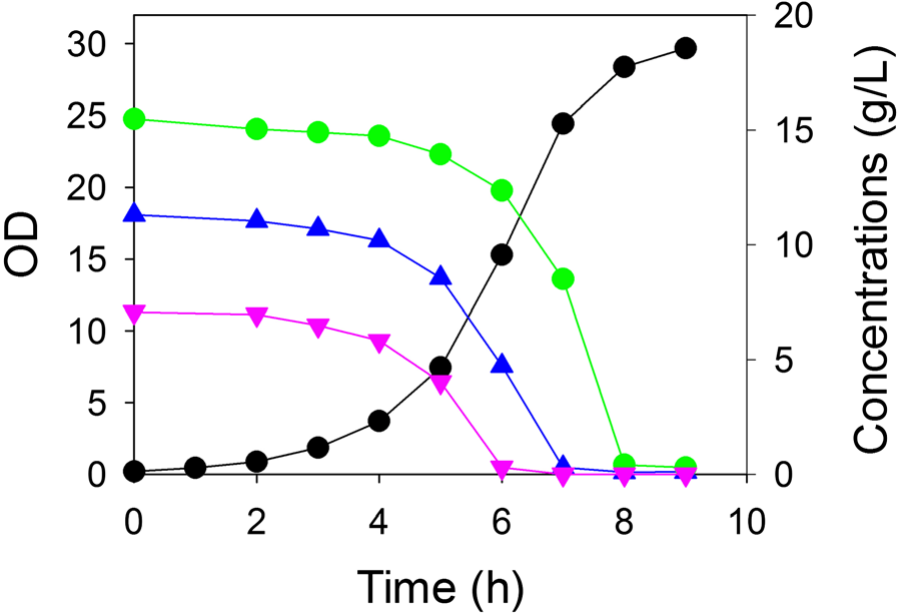
**Three strain growth in a controlled bioreactor containing a three sugar mixture (glucose, xylose and arabinose).** Growth (OD, *black circle*) of *E. coli* arabinose-selective strain ALS1370, xylose-selective strain ALS1371 and glucose-selective strain ALS1391 together in a medium containing three sugars: xylose (*blue triangle*), arabinose (*pink inverted triangle*) and glucose (*green circle*).

Because the preceding result used relatively low sugar concentrations, we repeated the study using the three-strain consortium in a controlled bioreactor with higher concentrations of the glucose, xylose and arabinose (Figure 3). In this case, the three sugars were again simultaneously consumed and to completion within 8 h, with arabinose exhausted first as its concentration was the lowest of the three sugars (7 g/L). The apparent growth rate of this consortium was 0.71 h^-1^, about 10% greater than observed in the comparatively uncontrolled shake flask experiment.

### Acetate degradation

**Figure 4:**
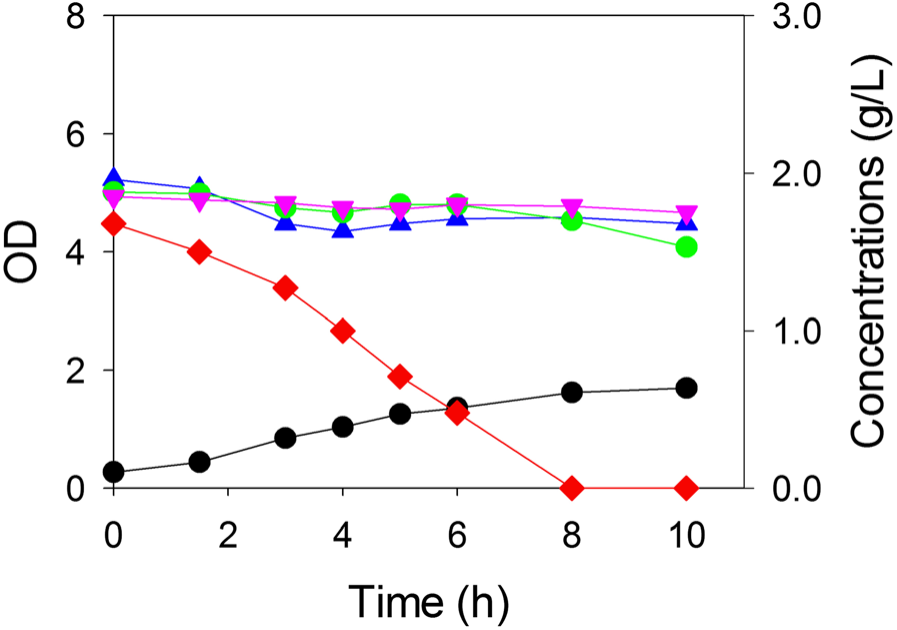
**Single strain growth on acetate in shake flasks containing a four-component mixture (acetate, glucose, xylose and arabinose).** Growth (OD, *black circle*) of *E. coli* acetate-selective strain ALS1392 in a medium containing acetate (*red diamond*) and three sugars: xylose (*blue triangle*), arabinose (*pink inverted triangle*) and glucose (*green circle*).

The substrate-selective approach should also be able to remove selectively an undesirable compound from a mixture. So, we constructed ALS1392 (C *ptsG manZ glk crr xylA araA*) which would not be able to consume any of the three sugars, but would be able to consume acetate. This strain was examined in shake flasks for its ability to consume acetate selectively from a mixture of glucose, xylose and arabinose. We first grew ALS1392 in a medium containing acetate alone, and in replicate experiments, the maximum specific growth rate was calculated to be 0.32 h^-1^. In the sugar mixture, ALS1392 grew with an essentially identical maximum specific growth rate of 0.35 h^-1^, and did not metabolize any of the sugars within the 6 h required for the exhaustion of acetate (Figure 4). These results demonstrate that acetate can be removed selectively from a synthetic three-sugar mixture, and that the presence of these three sugars at low concentrations does not affect cell growth rate.

### Controlled process to consume acetate then metabolize three sugars simultaneously

**Figure 5:**
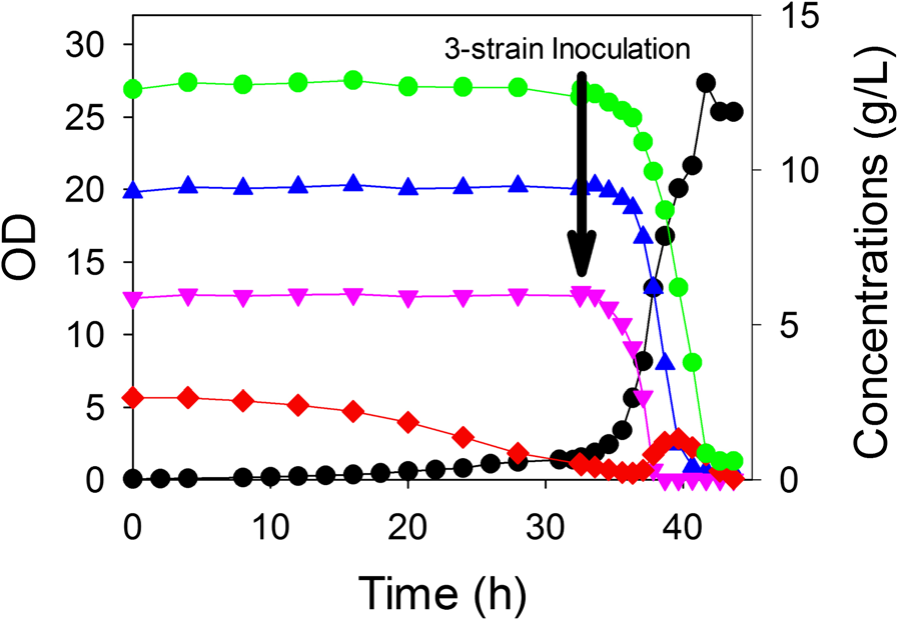
**Four strain growth in a controlled bioreactor containing a four-component mixture (acetate, glucose, xylose and arabinose).** First, the culture was inoculated with *E. coli* ALS1392. At 32.0 h as indicated, the culture was inoculated with a consortium of three strains: ALS1370, ALS1371 and ALS1391. Growth was measured as OD (*black circle*) in a medium containing acetate (*red diamond*) and three sugars: xylose (*blue triangle*), arabinose (*pink inverted triangle*) and glucose (*green circle*).

To simulate a process that might be used to generate a product from a biomass hydrolysate, we prepared a medium nominally containing 3 g/L acetate, 14 g/L glucose, 11 g/L xylose and 7 g/L arabinose. Initially, the culture was inoculated with ALS1392 which was expected to consume exclusively acetate. At 32 h when the acetate was nearly exhausted, the culture was inoculated with a mixture of the three sugar-consuming strains each at the same initial cell density of an OD of 0.1 in the bioreactor. As shown in Figure 5, ALS1392 indeed did consume exclusively acetate initially with a specific growth rate of 0.13 h^-1^. After inoculation with the three strains, glucose, xylose and arabinose were consumed within about 8 h, with some acetate being generated during this period.

**Figure 6:**
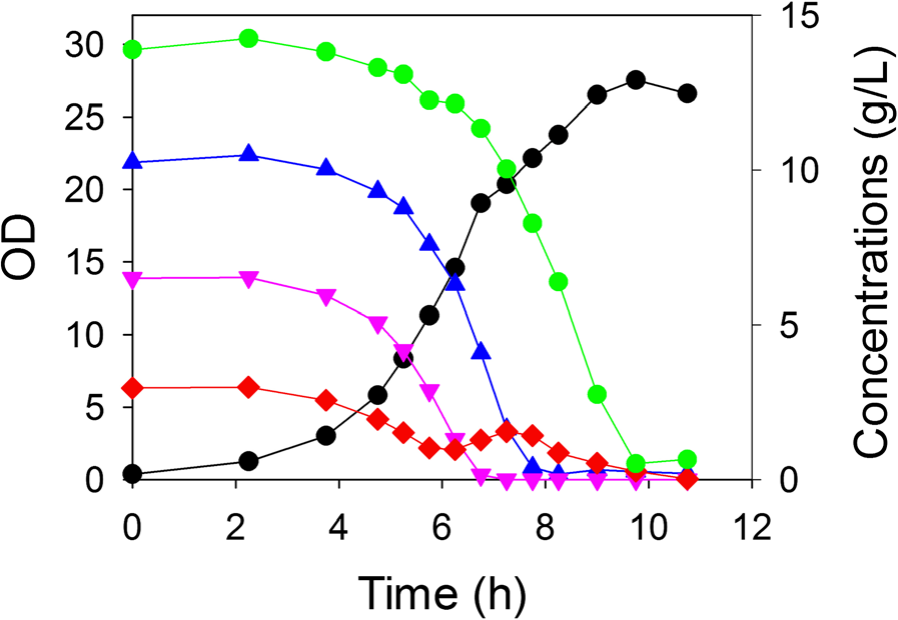
**Four strain growth in a controlled bioreactor containing a four-component mixture (acetate, glucose, xylose and arabinose).** The culture was inoculated simultaneously with *E. coli* ALS1370, ALS1371, ALS1391 and ALS1392. Growth was measured as OD (*black circle*) in a medium containing acetate (*red diamond*) and three sugars: xylose (*blue triangle*), arabinose (*pink inverted triangle*) and glucose (*green circle*).

Another approach that could be envisioned is the degradation of acetate in conjunction with sugar conversion. Such a process might be overall faster than a sequential process shown in Figure 5. We therefore repeated the process nominally containing 3 g/L acetate, 14 g/L glucose, 11 g/L xylose and 7 g/L arabinose. However, in this case the culture was simultaneously inoculated with *all four strains*, one that is unable to degrade any sugar and three which are selective for one of the sugars (but which could also consume acetate). As shown in Figure 6, the acetate, arabinose, xylose and glucose consumption occurred simultaneously. The overall process required just 10 h, which compares favorably to the same process without acetate requiring about 8 h (Figure 3). Similar to the results in which acetate and sugar consumption was sequential (Figure 5), a net accumulation of acetate was observed briefly during the period that glucose was being consumed.

## Discussion

In this study, a consortium of organisms was used to metabolize components of a sugar mixture containing the inhibitor acetate. First, acetate was selectively removed from the mixture using a strain of *E. coli* unable to consume any of the sugars. Then, three *E. coli* C knockout strains were simultaneously introduced into the sugar mixture, and each strain selectively utilized a single target sugar with minimal influence from the other strains or from the other nonmetabolizable sugars.

Acetate can be degraded selectively from a mixture containing glucose, xylose and arabinose. In a previous study, *E. coli* MG1655 derivatives were used to remove acetate selectively from a mixture of acetate, xylose and glucose [18]. Here we extend those results, introduce arabinose into the sugar mixture, and also examine the consumption of the three sugars. Interestingly, the C derivative ALS1392 used in the present study grew at a significantly greater growth rate on acetate than reported for the wild-type strain MG1655 (0.35 h^-1^ versus 0.22 h^-1^). Moreover, although slow sugar consumption by an acetate-selective strain of MG1655 was observed after acetate depletion [18], sugar consumption did not occur in the presence of acetate. In the current study we did not observe appreciable glucose consumption after 32 h. When it occurs, glucose degradation might occur as a result of a mutation or by the induction of an unknown gene. Any inadvertent sugar consumption by the acetate-consuming strain could be prevented merely by lysing these cells using any one of a variety of methods (i.e., a pH shift, a temperature increase, the use of an antibiotic). This approach might also permit the cell lysate to be an additional carbon source for the sugar-metabolizing consortium. Although the maximum specific growth rate of *E. coli* on acetate is less than one-half of the growth rate observed for any of the sugars, clearly the designation of acetate as a “toxic” compound is misleading. Interestingly, cultures involving all four strains accumulated acetate after the initial acetate had been largely depleted by ALS1392, but acetate was again soon exhausted before glucose. These results suggest that during this period acetate was being generated from sugars at a rate greater than the acetate-consuming cells were able to degrade. This brief period of acetate accumulation was quickly reversed as acetate-consuming cells were able to consume this substrate faster than it was being generated. Furthermore, all four of the strains are able to consume acetate, and it is unclear what portion of the consumption is due exclusively to ALS1392.

In this study in a controlled bioreactor, the acetate removal and sugar utilization were completed sequentially and in parallel. A two-phase, sequential batch mode of operation was conducted in order to ensure the absence of acetate and thus maximal sugar consumption during the second phase. However, since the acetate did not significantly affect sugar metabolism at the concentration used (3 g/L), the parallel process was ultimately able to consume the sugars much more quickly. An optimal process would be dictated by the specific concentrations of sugars and acetate and the extent to which the product formation rate is affected by acetate. The complete steps of acetate removal and sugar conversion might also be conducted simultaneously using some other operational mode such as a fed-batch process.

Although the growth rates of ALS1370 and ALS1371 remained above 0.7 h^-1^, arabinose and xylose respectively were both consumed 12–15% slower in these strains compared to wild-type C. This observation is consistent with the reduced rate of cyclic AMP (cAMP) synthesized in *crr* mutants. Specifically, in addition to hexose uptake, the PTS in *E. coli* is also involved in the regulation of adenylate cyclase (AC) activity. Upon binding of AC to the phosphorylated gene product of *crr*, the cell synthesizes cAMP, which in turn binds to the cAMP receptor protein (CRP) and induces catabolite-repressed genes [23]. Indeed, the cAMP-CRP complex transcriptionally activates more than a hundred genes and operons in *E. coli*[26,27], including arabinose uptake in concert with AraC via the *araBAD* system [28]. Reduced cAMP levels occur in an *E. coli crr* mutant [29], and in the present study the absence of IIA^Glc^ in ALS1370 and ALS1371 would similarly be expected to have reduced adenylate cyclase activity and cAMP concentration, potentially leading to reduced transcription of genes involved in arabinose and xylose metabolism. In *S. typhimurium*, *crr* mutants could not grow on several non-PTS compounds including xylose in the absence of externally supplied cAMP [30]. The absence of *crr* in *E. coli* and its associated effect on cAMP does not appear to have such a severe impact on *E. coli* growth on xylose or arabinose.

The growth rates of both ALS1371 (xylose-selective) and ALS1391 (glucose-selective) were reduced by over 20% in the three sugar mixture compared to a medium containing the single sugar (i.e., either xylose or glucose, respectively). No similar reduction in growth rate was observed in ALS1370 (arabinose-selective) on the sugar mixture compared to medium containing only arabinose. ALS1371 and ALS1391 share the characteristic of having an *araA* gene deletion, and moreover the sugar mixture contained arabinose whereas the single sugar medium for these two strains did not contain arabinose. Previous studies have reported that *E. coli* will consume arabinose first in a mixture of xylose and arabinose [31-33]. Moreover, arabinose directly represses the promoter for *xylA* through its binding to AraC [33]. Since only the *araA* gene was deleted in ALS1371 to eliminate arabinose consumption in our study, AraC remained available to repress xylose utilization when arabinose was present in the sugar mixture. The specific effect of arabinose was confirmed by comparing the growth rates of ALS1371 on two-sugar mixtures glucose-xylose and arabinose-xylose. Whereas ALS1371 attained a growth rate of about 0.71 h^-1^ on xylose alone, the growth rate of ALS1371 on the glucose-xylose mixture was 0.62 h^-1^. However, in either case the addition of arabinose further reduced growth rate: the growth rate of ALS1371 on arabinose-xylose was 15% lower than on xylose alone, while it was 13% lower on the three sugar mixture compared to on glucose-xylose. Surprisingly, we observed a similar arabinose-effect with ALS1391, which consumes only glucose in the presence of xylose and/or arabinose. Compared to ALS1391 growing on glucose alone, the growth rate on glucose-xylose was 19% lower, while the growth rate on glucose-arabinose was 44% lower. Although no previous study has similarly linked AraC and the glucose PTS, one explanation for the reduced growth rate of ALS1391 in the presence of arabinose compared to in a glucose-only medium is an analogous (i.e., transcriptional) mechanism in which AraC-arabinose represses the glucose PTS. Another potential explanation is that arabinose directly inhibits an enzyme of the glucose PTS. Any deleterious, specific effect of AraC could be removed by knocking out the *araC* gene instead of (or in addition to) the *araA* gene as a means to prevent arabinose consumption.

## Conclusions

This study is the first report of the truly *simultaneous* consumption of glucose, xylose, and arabinose in the presence of acetate. The removal of acetate is incorporated into the biological process, and indeed a portion of the acetate carbon became cellular carbon which could serve as a carbon source for sugar consumption, particularly when the acetate-selective cells lyse in the absence of a usable carbon source. Although not examined in this study, the rate of consumption of any one sugar could be further modulated independently simply by altering the relative population of the strains [17]. Another operational mode, such as a fed-batch process, would further benefit this process by preventing exposure to high concentrations of any of the substrates, while still permitting independent consumption. This approach, based on the *exclusion* of catabolic abilities instead of the enhancement of catabolic abilities, holds potential to be a new and effective microbial cell factory for the conversion of mixtures into bioproducts. We next plan to study the generation of products from sugar mixtures and actual hydrolysates, accomplished by applying additional metabolic engineering strategies to each member of the consortium.

## Methods

### Strains

*Escherichia coli* C (ATCC8739) was used in this study. Sugar-selective strains were the arabinose-selective strain ALS1370 (C *ptsG*763::(FRT) *glk*-726::(FRT) *manZ*743::(FRT) *crr*-746::(FRT) *xylA*748::(FRT)), which could not consume glucose or xylose, the xylose-selective strain ALS1371 (C *ptsG*763::(FRT) *glk*-726::(FRT) *manZ*743::(FRT) *crr*-746::(FRT) *araA*::(FRT)), which could not consume arabinose or glucose, and the glucose-selective strain ALS1391 (C *xylA*::(FRT) *araA*::(FRT)Kan), which could not consume arabinose or xylose. The acetate-selective strain ALS1392 (C *ptsG*763::(FRT) *glk*-726::(RT) *manZ*743::(FRT) *crr*-746::(FRT) *xylA*::(FRT)Kan *araA*::(FRT)) contained knockouts in all of the sugar-metabolizing genes and thus could not consume arabinose, glucose, or xylose. These strains were constructed by transducing ATCC8739 with the corresponding Keio (FRT)Kan deletions [34] and then curing the Kan(R) using the pCP20 plasmid, which contains a temperature-inducible FLP recombinase as well as a temperature-sensitive replicon [35].

### Growth conditions

The defined medium used in all shake flask experiments contained (per L): 26.6 g KH_2_PO_4_, 8.0 g (NH_4_)_2_HPO_4_, 1.2 g MgSO_4_·7H_2_O, 1.3 mg Zn(CH_3_COO)_2_·2H_2_O, 0.15 mg CuCl_2_·2H_2_O, 1.5 mg MnCl_2_·4H_2_O, 0.25 mg CoCl_2_·6H_2_O, 0.30 mg H_3_BO_3_, 0.25 mg Na_2_MoO_4_·2H_2_O, 10 mg Fe(III)citrate, 8.4 mg Na_2_EDTA·2H_2_O, 0.17 g citric acid, and 4.5 mg thiamine·HCl. For these experiments, D-(+)-glucose, D-(+)-xylose, L-(+)-arabinose, and/or acetate were added as carbon sources at a concentration of 2.25 g/L, and the pH adjusted initially to 7.0 using 30% NaOH. For experiments using a controlled bioreactor, the defined medium contained (per L): 13.3 g KH_2_PO_4_, 4.0 g (NH_4_)_2_HPO_4_, 1.2 g MgSO_4_·7H_2_O, 13.0 mg Zn(CH_3_COO)_2_·2H_2_O, 1.5 mg CuCl_2_·2H_2_O, 15.0 mg MnCl_2_·4H_2_O, 2.5 mg CoCl_2_·6H_2_O, 3.0 mg H_3_BO_3_, 2.5 mg Na_2_MoO_4_·2H_2_O, 100 mg Fe(III)citrate, 8.4 mg Na_2_EDTA·2H_2_O, 1.7 g citric acid, 4.5 mg thiamine·HCl, and three carbon sources 15.0 g glucose, 11.0 g xylose and 7 g arabinose. A study with four carbon sources used this same medium with additionally 6.9 g Na(CH_3_COO)·3H_2_O (equivalent to 3 g acetate). These media were adjusted to a pH of 7.0 using 30% NaOH.

### Cultures

Comparisons of *Escherichia coli* C, ALS1370, ALS1371, and ALS1391 were completed by first growing cells at 37°C with agitation of 250 rpm (19 mm pitch) in 125 mL shake flasks containing 20 mL defined medium with carbon substrate(s). When the OD of a culture reached approximately 1, 10 mL was transferred to a 500 mL shake flask containing 50 mL defined medium with carbon substrate(s) from which optical density was measured to determine growth rates. In multi-strain experiments, single strain cultures were briefly centrifuged (4°C, 3800 × g for 2 min), then resuspended with other strains in 10 mL defined medium to result in an effective OD of 1 for each of strain. Cultures were grown in duplicate.

Controlled batch processes at 1.0 L volume were carried out in a 2.5 L bioreactor (Bioflo 2000, New Brunswick Scientific Co. Edison, NJ, USA). Air or oxygen as necessary was sparged into the fermenter with the agitation set at 500 rpm to maintain the dissolved oxygen at above 40% saturation. The pH was controlled at 7.0 using 15% NaOH/20% KOH and 20% (v/v) H_2_SO_4_, and the temperature was controlled at 37°C. 3.0 g NH_4_Cl was added to the bioreactor when the OD reached 10 and again when the OD reached 20. Antifoam C (Sigma) was used as necessary to control foaming.

### Analyses

The OD measured at 600 nm (DU-650 spectrophotometer, Beckman Instruments, San Jose, CA) was used to monitor cell growth. Liquid chromatography with a refractive index detector was used to quantify acetate and sugars [36].

## Competing interests

The authors declare that they have no competing interests.

## Authors’ contributions

TX carried out the fermentations and acquired the data. ME and EA conceived the study. TX, ME and EA designed components of the study and interpreted the results. TX prepared the manuscript draft. All authors read and approved the final manuscript.
